# Association of Insurance Coverage With Adoption of Sleeve Gastrectomy vs Gastric Bypass for Patients Undergoing Bariatric Surgery

**DOI:** 10.1001/jamanetworkopen.2022.25964

**Published:** 2022-08-18

**Authors:** Ryan Howard, Edward C. Norton, Jie Yang, Jyothi Thumma, David E. Arterburn, Andrew Ryan, Dana Telem, Justin B. Dimick

**Affiliations:** 1 Department of Surgery, University of Michigan, Ann Arbor; 2 Center for Healthcare Outcomes and Policy, University of Michigan, Ann Arbor; 3 Department of Health Management and Policy, School of Public Health, University of Michigan, Ann Arbor; 4 Department of Economics, University of Michigan, Ann Arbor; 5 Kaiser Permanente Washington Health Research Institute, Seattle, Washington; 6 Center for Evaluating Health Reform, University of Michigan, Ann Arbor; 7 Division of Minimally Invasive Surgery, Department of Surgery, University of Michigan, Ann Arbor

## Abstract

**Importance:**

Instrumental variables can control for selection bias in observational research. However, valid instruments are challenging to identify.

**Objective:**

To evaluate regional variation in sleeve gastrectomy following insurance coverage implementation as an instrumental variable in comparative effectiveness research.

**Design, Setting, and Participants:**

This serial cross-sectional study included adult patients in a national Medicare claims database who underwent sleeve gastrectomy or Roux-en-Y gastric bypass from 2012 to 2017. Data analysis was performed from January to June 2021.

**Exposures:**

Laparoscopic sleeve gastrectomy and Roux-en-Y gastric bypass.

**Main Outcomes and Measures:**

The association of the instrumental variable with treatment (ie, undergoing sleeve gastrectomy), as well as mortality, complications, emergency department visits, hospitalization, reinterventions, and surgical revision.

**Results:**

A total of 76 077 patients underwent bariatric surgery, of whom 44 367 underwent sleeve gastrectomy (mean [SD] age, 56.9 [11.9] years; 32 559 [73.5%] women) and 31 710 underwent gastric bypass (mean (SD) age, 55.9 (11.8) years; 23 750 [74.9%] women). After insurance coverage initiation, there was substantial regional and temporal variation in adoption of sleeve gastrectomy. Prior-year state-level utilization of sleeve gastrectomy was highly associated with undergoing sleeve gastrectomy (Kleibergen-Paap Wald *F* statistic, 910.3). All but 2 patient characteristics (race and diagnosis of depression) were well-balanced between the top and bottom quartiles of the instrumental variable. Regarding 1-year outcomes, compared with patients undergoing gastric bypass, patients undergoing sleeve gastrectomy had a lower 1-year risk of mortality (0.9%; 95% CI, 0.8%-1.1% vs 1.7%; 95% CI, 1.3%-2.0%), complications (11.6%; 95% CI, 10.9%-12.3% vs 14.1%; 95% CI, 13.0%-15.3%), emergency department visits (48.3%; 95% CI, 46.9%-49.8% vs 53.6%; 95% CI, 52.3%-55.0%), hospitalization (23.4%; 95% CI, 22.4%-24.4% vs 26.5%; 95% CI, 25.1%-28.0%), and reinterventions (8.7%; 95% CI, 8.0%-9.4% vs 12.2%; 95% CI, 11.2%-13.3%). The risk of revision was not different between groups (0.6%; 95% CI, 0.3%-0.8% vs 0.4%; 95% CI, 0.3%-0.6%).

**Conclusions and Relevance:**

In this cross-sectional study of patients undergoing bariatric surgery, there was significant geographic variation in the use of sleeve gastrectomy following initiation of insurance coverage, which served as a strong instrument to compare 2 bariatric surgical procedures. This approach could be applied to other areas of health services research to serve as a complement to clinical trials.

## Key Points

**Question:**

Can the effects of national insurance coverage decisions serve as an instrumental variable in comparative effectiveness research?

**Findings:**

In this cross-sectional study of 76 077 patients who underwent sleeve gastrectomy and gastric bypass, initiation of insurance coverage for sleeve gastrectomy in 2012 was associated with significant temporal and regional practice variation that was associated with treatment and balanced characteristics between treatment arms, making it a strong instrumental variable.

**Meaning:**

These results suggest that initiation of insurance coverage for sleeve gastrectomy was associated with significant geographic and temporal variation, which served as a strong instrument to compare 2 bariatric surgical procedures.

## Teaser

This cross-sectional study compares demographic and insurance cover factors associated with the selection of sleeve gastrectomy or gastric bypass by time period and regional variation.

## Introduction

Randomized clinical trials are the criterion standard for establishing causal inference in comparative effectiveness research; however, logistic and financial barriers often limit their use in surgical research.^1,2^ While observational studies enable large-scale investigation of treatments in clinical settings, the results can be biased since treatment choice is often associated with patient characteristics (ie, selection bias) and the correlations demonstrated do not imply causation.^3^ A powerful approach to address the problem of selection bias in observational data is through the use of an instrumental variable.^4^ An instrumental variable is an exogenous factor that influences the likelihood of receiving a treatment without exerting any direct effect on the outcome of that treatment.^5,6,7^ By influencing treatment choice without directly affecting treatment outcome, a valid instrumental variable can effectively randomize treatment for a subset of patients, which allows for estimation of unbiased treatment effects.^8^

Although effective in their ability to reduce bias, instrumental variables can be difficult to identify.^9^ Specifically, a valid instrument relies on the assumption that it is associated with receiving a given treatment, but that it is neither directly nor indirectly (except through treatment) associated with the outcome of that treatment.^10^ Given these requirements, strong instruments typically rely on natural sources of variation.^11^ Large-scale policy decisions often create significant geographic variation.^12^ For example, in 2012 the US Centers for Medicare and Medicaid Services (CMS) decided that “effective for services performed on and after June 27, 2012, Medicare Administrative Contractors (MAC) acting within their respective jurisdictions may determine coverage of stand-alone laparoscopic sleeve gastrectomy for the treatment of comorbid conditions related to obesity in Medicare beneficiaries.”^13^ This decision had the potential to create significant geographic and temporal variation in the utilization of sleeve gastrectomy, providing the opportunity to use this variation as an instrumental variable.

Within this context, we sought to understand how variation following insurance coverage implementation could be used as an instrumental variable in surgical research. As a case study, we selected regional variation in utilization of laparoscopic sleeve gastrectomy and laparoscopic gastric bypass among Medicare beneficiaries following insurance coverage implementation. We primarily assessed the strength and validity of the instrumental variable and secondarily applied this instrumental variable to evaluate patient outcomes. We hypothesized that variation in the utilization of these procedures would create a quasi-experimental state that could serve as a valid instrumental variable in analyzing the comparative safety of these 2 common surgical procedures. This could have implications for the use of instrumental variables analysis in other instances of practice variation as the result of policy decisions.^14,15^

## Methods

### Data Source and Study Population

This study utilized 100% fee-for-service Medicare claims (Part A, Part B, outpatient, and home health agency) for patients undergoing laparoscopic sleeve gastrectomy or laparoscopic Roux-en-Y gastric bypass from January 1, 2012, to December 31, 2017. Eligible cases were identified using *Current Procedural Terminology* (CPT) codes 43775, 43644, and 43645 with matching *International Classification of Diseases, Ninth Revision (ICD-9)* and *International Statistical Classification of Diseases and Related Health Problems, Tenth Revision (ICD-10)* diagnosis codes for severe obesity. Urgent or emergent operations were excluded. Patients were excluded if their Medicare entitlement was due to end-stage kidney disease or if they had a diagnosis code associated with gastric or small bowel cancer, because these patients have a far more severe underlying comorbidity burden.

### Instrumental Variable Definition

The instrumental variable employed in this study was state-level sleeve gastrectomy utilization among Medicare beneficiaries in the prior year (relative to gastric bypass).^16,17^ The rationale for this instrumental variable was as follows. CMS divides the US into Medicare Administrative Contractor (MAC) jurisdictions. A MAC is a private health care insurer that is responsible for administering Medicare Part A and Part B claims for their jurisdiction. In June 2012, there were 14 such jurisdictions, and CMS issued a National Coverage Determination (NCD) stating that “effective for services performed on and after June 27, 2012, Medicare Administrative Contractors (MAC) acting within their respective jurisdictions may determine coverage of stand-alone laparoscopic sleeve gastrectomy for the treatment of comorbid conditions related to obesity in Medicare beneficiaries.”^13^ Although coverage decisions by individual MACs could not be evaluated, we hypothesized that this decision would result in both geographic and temporal variation in the utilization of sleeve gastrectomy. This variation would then create the opportunity for a natural experiment in which treatment choice could be projected to a large extent based on whether a patient lived in a region with high or low sleeve gastrectomy utilization and would therefore be pseudorandom. This is similar to other studies that have successfully used variation in geographic factors as an instrumental variable.^7^ Because coverage determinations at the level of the MAC jurisdiction are not publicly reported, we chose state-level sleeve gastrectomy utilization as the instrumental variable.

### Outcomes

The primary outcomes of this study were the strength and validity of the instrumental variable. These were assessed using the Kleibergen-Paap Wald *F* statistic and the balance of patient characteristics using the instrumental variable (as defined in Statistical Analysis).

In addition to evaluating the strength and validity of the instrumental variable in this study, we secondarily evaluated the following clinical outcomes: mortality, complications, emergency department (ED) visits, hospitalization, reinterventions, and revisions 1 year after operation. Complications associated with bariatric surgery were defined using *ICD-9* or *ICD-10* diagnosis and procedures codes and included splenic, hemorrhagic, anastomotic, wound-related, obstruction-related, pulmonary, cardiac, neurological, genitourinary, thromboembolic, shock, and unexpected reoperations. Reinterventions were identified using appropriate CPT codes and grouped into 5 categories: revision, reoperation, enteral access, vascular access, and other interventions. Revisions included any operation that involved modifying the index bariatric procedure. Reoperations included any abdominal operation potentially related to the index bariatric procedure but not directly affecting bariatric physiology. Reoperations within the first 30 days of the index bariatric procedure were excluded as these may represent delayed claims for procedures performed concurrently with the index operations.^16,18^ ED utilization was identified using a revenue center code algorithm previously described by the Research Data Assistance Center.^19^ ED utilization resulting in hospital admission was categorized as hospitalization only.

### Statistical Analysis

To determine whether a variable serves as a valid instrument, it must first be associated with treatment selection.^4^ We evaluated this condition by calculating the Kleibergen-Paap Wald *F* statistic for previous-year sleeve gastrectomy rate and current year treatment (ie, undergoing sleeve gastrectomy). An *F* statistic greater than 10 is generally considered to be a strong instrument.^20^ Second, the instrument must not be associated with the outcome except through treatment. While this condition cannot be empirically proven, it can be evaluated on both a theoretical basis and by examining the balance of patient covariates stratified by the instrument. Lagged local treatment patterns are believed to satisfy this condition because they reflect patient comorbidities and care decisions from a previous time period among a different set of patients.^6^ Moreover, a regional trend in a previous year is likely to influence decisions made during the current year.

Regarding balance, similar to how the 2 groups of a randomized controlled trial are ideally similar to each other, patient characteristics when stratified by the instrument should be similar as well. For example, although the prevalence of diabetes is often much higher among patients undergoing gastric bypass, we would not expect substantial differences in diabetes when stratifying patients based on the prior year’s sleeve gastrectomy rate. Therefore, we calculated the balance of patient-level covariates at the actual treatment level and at the top and bottom quartiles of the instrument.

Simple logistic regression models and instrumental variable models were used to estimate the adjusted absolute risk difference of each outcome. The instrumental variable model used a 2-stage residual inclusion estimation method.^21^ In the first stage, multivariable logistic regression was performed to estimate the likelihood that a patient would undergo sleeve gastrectomy while adjusting for the following covariates: previous-year state-level sleeve gastrectomy rate (the instrumental variable), age, sex, race and ethnicity, comorbidities, and year of surgery (eTable 1 in the Supplement). Race and ethnicity were included since they have previously been found to be associated with bariatric surgical outcomes, and were identified using identifiers in the Medicare claims database. In the second stage, a logistic regression model was constructed to estimate the absolute risk difference for each outcome while adjusting for the following covariates: treatment (sleeve gastrectomy vs gastric bypass), age, sex, race and ethnicity, comorbidities, year of surgery, and importantly, residuals from the first-stage regression model, which represent unobserved confounding associated with treatment choice.^22^ Put another way, this approach “accounts for” selection bias using the instrumental variable. The augmented Durbin-Wu-Hausman test was used to assess whether endogeneity was present for any outcomes.^23,24^ We also performed a sensitivity analysis in which the instrumental variable was lagged by 2 years instead of 1 (eTable 2 in the Supplement).

All statistical tests were performed using SAS version 9.4 (SAS Institute Inc) and STATA version 15.1 (StataCorp Inc). Tests were 2-sided and significance was set at *P* < .05. Robust standard errors were used to account for state-level heteroscedasticity. Data analysis was performed from January to June 2021. This secondary analysis of deidentified administrative claims data was determined to be exempt from regulation by the University of Michigan institutional review board. This study followed the Strengthening the Reporting of Observational Studies in Epidemiology (STROBE) reporting guideline.

## Results

From 2012 to 2017, 76 077 patients underwent surgery, of which 44 367 (58.3%) patients underwent sleeve gastrectomy and 31 710 (41.7%) patients underwent gastric bypass (Table 1). The mean (SD) age of patients undergoing sleeve gastrectomy was 56.9 (11.9) years, and 32 559 (73.4%) were women. The mean age of patients undergoing gastric bypass was 55.9 (11.8) years, and 23 750 (74.9%) were women.

**Table 1.** Covariate Balance Grouped by Actual Treatment and Grouped by Top and Bottom Quartiles of Prior-Year Sleeve Gastrectomy Utilization

Characteristic	Actual treatment				Prior year SG utilization rate (IV)			
	No. (%)		Standardized difference, %^a^	*P* value	No. (%)		Standardized difference, %^a^	*P* value
	SG (N = 44 367)	GB (N = 31 710)			4th quartile (N = 20 027)	1st quartile (N = 18 440)		
Sleeve gastrectomy	44 367 (100)	31 710 (0)	NA	NA	13 902 (69.4)	8821 (47.8)	44.9	<.001
Age, y								
≤55	18 486 (41.7)	14 157 (44.7)	6.0	<.001	8620 (43.0)	7780 (42.2)	1.1	.092
55-65	10 649 (24.0)	7980 (25.2)	2.7	<.001	24.83 (24.5)	4579 (24.8)	0.4	.41
66-75	14 777 (33.3)	9383 (29.6)	8.0	<.001	6350 (31.7)	5946 (32.3)	1.1	.26
>75	455 (1.0)	190 (0.6)	4.8	<.001	156 (0.8)	135 (0.7)	1.4	.60
Year of surgery								
2012	515 (1.2)	7841 (24.7)	75.0	<.001	2161 (10.8)	2102 (11.4)	1.9	.057
2013	6312 (14.2)	6045 (19.1)	13.0	<.001	2944 (14.7)	2557 (13.9)	2.4	.02
2014	8355 (18.8)	5193 (16.4)	6.5	<.001	4023 (20.1)	3363 (18.2)	4.7	<.001
2015	9523 (21.5)	4711 (14.9)	17.2	<.001	3653 (18.2)	3616 (19.6)	3.5	<.001
2016	9707 (21.9)	4085 (12.9)	23.9	<.001	3653 (18.2)	3506 (19.0)	2.0	.05
2017	9955 (22.4)	3835 (12.1)	27.6	<.001	3593 (17.9)	3296 (17.9)	0.2	.86
Women	32 559 (73.4)	23 750 (74.9)	3.5	<.001	14 735 (73.6)	13 826 (75.0)	3.4	.002
Race and ethnicity								
Asian	114 (0.3)	94 (0.3)	0.8	.30	40 (0.2)	44 (0.2)	0.8	0.41
Black	7818 (17.6)	4890 (15.4)	5.9	<.001	3602 (18.0)	2778 (15.1)	7.9	<.001
Hispanic	1471 (3.3)	955 (3.0)	1.7	.02	721 (3.6)	265 (1.4)	13.8	<.001
American Indian	254 (0.6)	231 (0.7)	1.9	.008	69 (0.3)	175 (1.0)	7.6	<.001
White	33 747 (76.1)	24 961 (78.7)	6.4	<.001	15 213 (76.0)	14 862 (80.6)	11.3	<.001
Other	381 (0.9)	268 (0.9)	0.2	.84	183 (0.9)	118 (0.6)	3.1	.002
Unknown	582 (1.3)	311 (1.0)	3.1	<.001	199 (1.0)	198 (1.1)	0.8	.44
Comorbidities								
Hypertension	33 800 (76.2)	24 369 (76.9)	1.6	.03	15 350 (76.7)	14 138 (76.7)	0.1	.96
Diabetes without chronic complications	16 541 (37.3)	14 439 (45.5)	16.8	<.001	8230 (41.1)	7520 (40.8)	0.6	.53
Depression	11 373 (25.6)	9442 (29.8)	9.2	<.001	5167 (25.8)	5628 (30.5)	10.5	<.001
Chronic pulmonary disease	11 497 (25.9)	8884 (28.0)	4.7	<.001	5189 (25.9)	5012 (27.2)	2.9	.005
Hypothyroidism	7901 (17.8)	5618 (17.7)	0.2	.74	3410 (17.0)	3472 (18.8)	4.7	<.001
Liver disease	5623 (12.7)	4591 (14.5)	5.3	<.001	2344 (11.7)	2275 (12.3)	2.0	.06
Psychoses	2718 (6.1)	2458 (7.8)	6.4	<.001	1216 (6.1)	1342 (7.3)	4.8	<.001
Deficiency anemias	3064 (6.9)	2213 (7.0)	0.3	.70	1407 (7.0)	1192 (6.5)	2.2	.03
Diabetes with chronic complications	3884 (8.8)	3218 (10.2)	4.8	<.001	1752 (8.8)	1916 (10.4)	5.6	<.001
Fluid and electrolyte disorders	2606 (5.9)	2065 (6.5)	2.7	<.001	1194 (6.0)	1168 (6.3)	1.6	.13
Congestive heart failure	2869 (6.5)	1969 (6.2)	1.1	.15	1260 (6.3)	1244 (6.8)	1.8	.07
Renal failure	3833 (8.6)	2250 (7.1)	5.7	<.001	1530 (7.6)	1591 (8.6)	3.6	<.001
Other neurological disorders	2373 (5.4)	1744 (5.5)	0.7	.36	1010 (5.0)	1197 (6.5)	6.2	<.001
Rheumatoid arthritis	1977 (4.5)	1158 (3.7)	4.1	<.001	828 (4.1)	756 (4.1)	0.2	.86
Peripheral vascular disease	767 (1.7)	622 (2.0)	1.7	.02	357 (1.8)	364 (2.0)	1.4	.17
Pulmonary circulation disease	386 (0.9)	449 (1.4)	5.1	<.001	201 (1.0)	236 (1.3)	2.6	.01
Valvular disease	853 (1.9)	570 (1.8)	0.9	.21	389 (1.9)	330 (1.8)	1.1	.27
Coagulopathy	427 (1.0)	308 (1.0)	0.1	.90	187 (0.9)	187 (1.0)	0.8	.42
Weight loss	127 (0.3)	137 (0.4)	2.4	.001	86 (0.4)	62 (0.3)	1.5	.14
Paralysis	240 (0.5)	157 (0.5)	0.6	.39	91 (0.5)	100 (0.5)	1.3	.22
Solid tumor without metastasis	115 (0.3)	71 (0.2)	0.7	.33	42 (0.2)	51 (0.3)	1.4	.18
Chronic blood loss anemia	54 (0.1)	64 (0.2)	2.0	.006	28 (0.1)	29 (0.2)	0.5	.66
Lymphoma	60 (0.1)	35 (0.1)	0.7	.34	32 (0.2)	22 (0.1)	1.1	.29
Acquired immune deficiency syndrome	65 (0.2)	30 (0.1)	1.5	.046	23 (0.1)	17 (0.1)	0.7	.49

Abbreviations: GB, gastric bypass; IV, instrumental variable; SG, sleeve gastrectomy.

^a^ Standardized difference equals the mean difference divided by the pooled standard deviation.

Utilization of sleeve gastrectomy increased during the study period from 515 procedures performed in 2012 to 9955 procedures performed in 2017. This represented an increase in the annual proportion of sleeve gastrectomy from 6.2% in 2012 to 72.2% in 2017. There was substantial regional and temporal variation in the increase of sleeve gastrectomy (Figure). Among a representative sample of the 10 largest states, the largest increase occurred in New Jersey, where utilization of sleeve gastrectomy increased from 7.9% in 2012 to 92.8% in 2017, and the smallest increase occurred in Ohio, where utilization of sleeve gastrectomy increased from 10.9% in 2012 to 63.2% in 2017.

**Figure.**
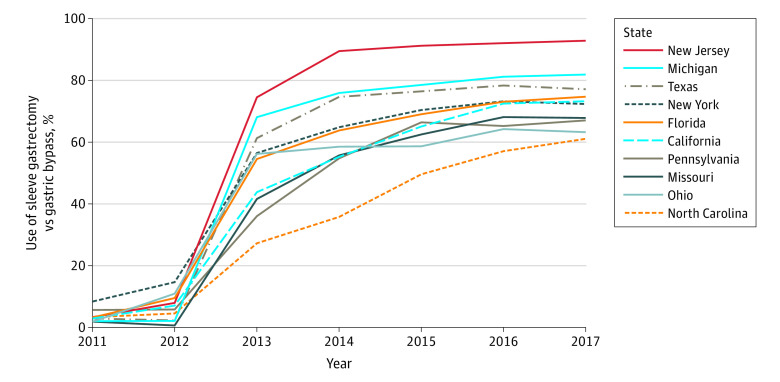
Change in Utilization of Sleeve Gastrectomy Among 10 Largest States From 2011-2017

Prior-year sleeve gastrectomy rate was highly associated with undergoing sleeve gastrectomy as evidenced by a Kleibergen-Paap Wald *F* statistic of 910.3. There were 20 027 (26.3%) patients in the bottom quartile and 18 440 (24.2%) in the top quartile of the instrumental variable. When considering the covariate balance between actual treatment groups and between the top and bottom quartiles of the instrumental variable, there was a significant reduction in covariate imbalance using the instrument (Table 1). For example, a standardized difference of 16.8% in the prevalence of diabetes between actual treatment groups (sleeve gastrectomy, 16 541 participants [37.3%] vs gastric bypass, 14 439 participants [45.5%]) was reduced to 0.6% using the instrument (8230 participants [41.1%] vs 7520 participants [40.8%]). After implementation of the instrument, race (11.3% for White race) and depression (10.5%) were the only covariates with standardized differences above 10%.

Analysis using an instrumental variable revealed that compared with patients undergoing gastric bypass, patients undergoing sleeve gastrectomy had a lower risk of mortality (0.9%; 95% CI, 0.8%-1.1% vs 1.7%; 95% CI, 1.3%-2.0%), complications (11.6%; 95% CI, 10.9%-12.3% vs 14.1%; 95% CI, 13.0%-15.3%), ED visits (48.3%; 95% CI, 46.9%-49.8% vs 53.6%; 95% CI, 52.3%-55.0%), hospitalization (23.4%; 95% CI, 22.4%-24.4% vs 26.5%; 95% CI, 25.1%-28.0%), and reinterventions (8.7%; 95% CI, 8.0%-9.4% vs 12.2%; 95% CI, 11.2%-13.3%). The risk of revision was not significantly different between groups (0.6%; 95% CI, 0.3%-0.8% vs 0.4%; 95% CI, 0.3%-0.6%). Finally, in comparing outcomes modeled using an instrumental variable with outcomes modeled using simple logistic regression, the augmented Durbin-Wu-Hausman test of endogeneity demonstrated that unobserved confounding was present for hospitalization and ED utilization (Table 2).

**Table 2.** Instrumental Variables and Simple Logistic Regression Models for Primary Outcomes

Outcome	Simple logistic regression, estimate (95% CI)^a^		Instrumental variables analysis, estimate (95% CI)^b^		*P* value for endogeneity^c^
	Sleeve	Bypass	Sleeve	Bypass	
Mortality	0.98 (0.88-1.08)	1.53 (1.41-1.65)	0.92 (0.80-1.05)	1.68 (1.33-2.04)	.28
Complications	11.17 (10.74-11.60)	14.74 (14.16-15.32)	11.6 (10.87-12.31)	14.13 (12.95-15.30)	.23
ED utilization	47.46 (46.57-48.36)	54.81 (54.10-55.52)	48.32 (46.88-49.78)	53.62 (52.30-54.95)	.05
Hospitalization	22.41 (21.65-23.18)	27.98 (2.725-28.70)	23.38 (22.39-24.38)	26.53 (25.10-27.96)	.008
Reintervention	8.55 (8.20-8.91)	12.45 (11.94-12.96)	8.68 (7.99-9.38)	12.21 (11.18-13.25)	.61
Revision	0.43 (0.34-0.51)	0.52 (0.43-0.61)	0.55 (0.30-0.81)	0.41 (0.26-0.55)	.15

Abbreviation: ED, emergency department.

^a^ Covariates included in the logistic regression model were patient age, sex, race and ethnicity, comorbidities, and year of surgery.

^b^ 2-stage residual inclusion estimation method wherein the first stage was a multivariable logistic regression model to estimate the likelihood of undergoing sleeve gastrectomy (covariates included previous-year state-level sleeve gastrectomy rate [the instrumental variable], age, sex, race and ethnicity, comorbidities, and year of surgery) and the second stage was a multivariable logistic regression model to estimate the absolute risk difference for each outcome (covariates included treatment, age, sex, race and ethnicity, comorbidities, year of surgery, and residuals from the first-stage regression model).

^c^ Endogeneity evaluated using the augmented Durbin-Wu-Hausman test. *P* ≤ .05 indicates the presence of endogeneity (ie, unobserved confounding) in the simple logistic regression model.

## Discussion

This study sought to understand whether geographic variation in the use of sleeve gastrectomy following implementation of insurance coverage could be used as an instrumental variable in analysis of observational data. We found that after CMS established Medicare coverage for sleeve gastrectomy in 2012, there was significant variation in sleeve gastrectomy utilization over time and between states. We also found that state-level utilization of laparoscopic sleeve gastrectomy in the prior year was highly associated with current-year treatment choice. Analysis using this source of variation as an instrumental variable subsequently produced results comparable with those of a logistic regression and revealed that patients undergoing sleeve gastrectomy are at lower risk of adverse outcomes compared with patients undergoing gastric bypass, which is consistent with prior studies.^16^ Given that geographic and temporal variation exists in other surgical procedures as the result of policy decisions and implementation, these findings may inform the use of instrumental variables analysis in surgical outcomes research.

These results suggest that geographic and temporal variation can serve as a strong instrument to account for bias in observational data. First, we found that our instrument—prior-year utilization rate of sleeve gastrectomy at the state level—was associated with treatment choice, as reflected by a Kleibergen-Paap-Wald *F* statistic of 910.30, which was well above the generally accepted threshold of 10 or greater for a strong instrument.^20,25,26^ This indicates that a patient’s likelihood of undergoing sleeve gastrectomy is associated with how widely used that operation was in their state in the prior year. Second, we found that assignment of patients to either treatment based on the instrument resulted in 2 cohorts with similar characteristics. The relatively large differences in age and comorbidities prior to application of the instrumental variable highlight the selection bias present in these data. For example, the older age and increased prevalence of comorbidities such as kidney failure among patients undergoing sleeve gastrectomy reflect the preferential use of this procedure in high-risk patients, because it is more well-tolerated than gastric bypass.^27,28^ Similarly, gastric bypass is often the procedure of choice for patients with obesity with concomitant diabetes because it may afford superior diabetes resolution compared with sleeve gastrectomy.^29^ However, after accounting for treatment assignment based in the instrumental variable, the differences in these characteristics between groups became negligible.

When both of these conditions are met, an instrument is considered valid and generates a condition of pseudo-randomization in that it influences a patient’s likelihood to undergo a particular treatment more or less by chance. Applying this to the current study, a patient who just happens to live in a region with high sleeve gastrectomy utilization is more likely to get a sleeve gastrectomy, and a patient who just happens to live in a region with low sleeve gastrectomy utilization is less likely to get a sleeve gastrectomy. However, it is important to note that although selection bias is clearly present in these 2 groups, the results of the instrumental variable analysis are overall similar to the results of the simple logistic regression model. This suggests that the bias present may have been minimal, or that the logistic regression model sufficiently controlled for these differences. Additionally, it is also possible that confounding was still present in the instrumental variable analysis, although the conditions met by our instrument provide reassurance that this would have been minimal.

Given that there is substantial geographic variation in surgical management in the US, the approach explored in the current study may have broad applicability. For example, women with breast cancer can be nearly 4-fold more likely to undergo breast-conserving surgery depending on where they live.^30^ Similarly, utilization of minimally invasive surgery, emergency general surgery, resection for common surgical diseases, and ambulatory surgery have been shown to vary many-fold throughout the US.^31,32,33,34^ To the extent that a patient’s likelihood of receiving a given treatment depends largely on where they live, this variation may be used as an instrumental variable in any number of surgical conditions. For example, regional variation in surgical approach has been used to explore the effects of open vs laparoscopic surgery on outcomes after colectomy.^35,36^

These results may have implications for health care policy. Although health care quality improvement requires rigorous evaluation of new policies, implementation of these policies often occurs without consideration of how they can be evaluated. In the current study, it just so happened that the Medicare coverage determination in 2012 resulted in a high degree of variation in utilization of sleeve gastrectomy. This allowed for pseudorandomization through the application of instrumental variables analysis, which strengthens the estimation of treatment effects. There have been other instances where variable application of new health care policy has enabled more rigorous evaluation, such as asynchronous implementation of Medicaid expansion by individual states.^14,15^ Therefore, policy design which deliberately, even if only temporarily, maximizes both regional and geographic variation may enable more rigorous evaluation of the results of new policy.

### Limitations

This study had several limitations. First, this study analyzed the strength and validity of an instrumental variable in a specific instance, namely, utilization of sleeve gastrectomy. It is likely that this represents a rather fortuitous instance and that finding similar instrumental variables in other specialties may be more difficult. Nevertheless, similar utilization of regional and temporal variation as an instrumental variable has been applied to colorectal surgery as described above. Another limitation is that even though the use of an instrumental variable allows for causal inference, the results of instrumental variables analysis only apply to patients whose treatment depends on the instrument.^8,37,38^ For example, patients who have a very strong indication for gastric bypass over sleeve gastrectomy derive little to no influence on their treatment from prior-year sleeve gastrectomy utilization. Therefore, the results of a study should be interpreted as applying only to those patients for which the instrumental variable likely played a role in treatment selection.^39^ Another limitation of this study is that despite use of an instrumental variable that we and others have successfully employed in previous work, imbalance persisted between groups, specifically with regards to patient race. Garabedian et al^10^ previously identified that factors such as urban vs rural and socioeconomic status were correlated with regional variation in surgical procedures and thereby may introduce bias into instrumental variables analysis. Although beyond the scope of the current study, additional work is needed to clarify how variations in socioeconomic status can be sufficiently accounted for in this type of analysis. Additionally, while lagged local treatment patterns have been used in other instrumental variable analyses, it is possible that current treatment patterns may serve as an equally or more valid instrumental variable. Indeed, this approach has been used to investigate surgical outcomes. Future methodologic work comparing the validity of various instruments could further inform the most appropriate way to rigorously evaluate real-world practice and outcomes. Finally, it is important to note that this study used regional variation in sleeve gastrectomy as an instrumental variable rather than regional variation in insurance coverage itself. While this nevertheless served as a strong instrument and was used since local coverage decisions are not publicly reported, it should be noted that use of policy decisions themselves have the potential to serve as an even strong instrument in these kinds of analyses.

## Conclusions

In this retrospective observational cross-sectional study, there was substantial geographic and temporal variation in utilization of sleeve gastrectomy following insurance coverage implementation which served as a strong and valid instrument in comparing the effectiveness of alternative bariatric surgical procedures. Insofar as such variation is not unique to bariatric surgery, this approach could be applied to other areas of health services research to generate rich clinical results that serve as an important complement to clinical trials.

## Notes

Supplement. **eTable 1.** First Stage of Instrumental Variable Analysis **eTable 2.** Results of a Sensitivity Analysis in Which the Instrumental Variable Is Utilization of Sleeve Gastrectomy in the 2 Years Prior to Treatment
