# Supplementary material for: Association of Insurance Coverage With Adoption of Sleeve Gastrectomy vs Gastric Bypass for Patients Undergoing Bariatric Surgery
Source: JAMA Netw Open. 2022 Aug 18;5(8):e2225964. doi: 10.1001/jamanetworkopen.2022.25964 (PMC9389353; doi:10.1001/jamanetworkopen.2022.25964)
Supplement: Supplement. — eTable 1. First Stage of Instrumental Variable Analysis eTable 2. Results of a Sensitivity Analysis in Which the Instrumental Variable Is Utilization of Sleeve Gastrectomy in the 2 Years Prior to Treatment [file jamanetwopen-e2225964-s001.pdf]

Supplemental Online Content

Howard R, Norton EC, Yang J, et al. Association of insurance coverage with adoption of sleeve gastrectomy vs gastric bypass for patients undergoing bariatric surgery. *JAMA Netw Open*. 2022;5(8):e2225964. doi:10.1001/jamanetworkopen.2022.25964

eTable 1. First stage of instrumental variable analysis

eTable 2. Results of a sensitivity analysis in which the instrumental variable is utilization of sleeve gastrectomy in the 2 years prior to treatment

This supplemental material has been provided by the authors to give readers additional information about their work.

© 2022 Howard R et al. *JAMA Network Open.*

eTable 1. First stage of instrumental variable analysis.

	β	95% CI	SE	Z-Stat	P
Prior-Year Sleeve Gastrectomy Use	3.919	3.433, 4.405	0.248	15.8	<.001
Age	0.004	0.001, 0.009	0.002	2.51	0.012
Female	-0.090	-0.135, -0.045	0.023	-3.9	<.001
Race/Ethnicity (ref: White)					
Black	0.189	0.100, 0.279	0.046	4.15	<.001
Other	0.075	-0.033, 0.182	0.055	1.36	0.174
Year of Surgery (ref: 2012)					
2013	2.638	2.235, 3.042	0.206	12.81	<.001
2014	1.352	0.970, 1.734	0.195	6.94	<.001
2015	0.879	0.458, 1.300	0.215	4.09	<.001
2016	0.812	0.376, 1.248	0.223	3.65	<.001
2017	0.768	0.318, 1.217	0.230	3.34	0.001
Comorbidities					
Congestive heart failure	0.107	-0.011, 0 .225	0.060	1.77	0.076
Valvular disease	0.091	-0.152, 0.334	0.124	0.73	0.463
Pulmonary circulation disease	-0.124	-0.354, 0.105	0.117	-1.06	0.290
Peripheral vascular disease	0.001	-0.185, 0.186	0.095	0.01	0.994
Hypertension	-0.116	-0.200, -0.032	0.043	-2.71	0.007
Paralysis	0.315	-0.068, 0.698	0.195	1.61	0.107
Other neurological disorders	0.090	-0.070, 0 .250	0.082	1.10	0.271
Chronic pulmonary disease	-0.073	-0.120, -0.026	0.024	-3.03	0.002
Diabetes without chronic complications	-0.334	-0.403, -0.264	0.035	-9.45	<.001
Diabetes with chronic complications	-0.58	-0.702, -0.459	0.062	-9.40	<.001
Hypothyroidism	0.045	-0.026, 0.115	0.036	1.24	0.213
Renal failure	0.361	0.232, 0.490	0.066	5.49	<.001
Liver disease	-0.146	-0.383, 0.088	0.120	-1.22	0.222
Acquired immune deficiency syndrome	-0.022	-0.911, 0.868	0.454	-0.05	0.962
Lymphoma	0.093	-0.627, 0.812	0.367	0.25	0.801
Solid tumor w/out metastasis	-0.222	-0.698, 0.253	0.757	-0.91	0.360
Rheumatoid arthritis	0.272	0.142, 0.403	0.243	4.09	<.001
Coagulopathy	0.120	-0.118, 0.359	0.066	0.99	0.323
Weight loss	-0.611	0.003, 0.295	0.122	-2.64	0.046
Fluid and electrolyte disorders	-0.080	-0.198, 0.036	0.059	-1.35	0.178
Chronic blood loss anemia	-0.513	-1.141, 0.113	0.32	-1.6	0.109
Deficiency Anemias	-0.025	-0.188, 0.138	0.083	-0.30	0.763
Psychoses	-0.210	-0.337, -0.082	0.065	-3.23	0.001
Depression	-0.097	-0.177, -0.016	0.041	-2.36	0.018

Supplemental Table 1 Legend: Results of a logistic regression with outcome being undergoing sleeve gastrectomy. Prior-year sleeve gastrectomy use is the instrumental variable.

© 2022 Howard R et al. *JAMA Network Open.*

eTable 2. Results of a Sensitivity Analysis in Which the Instrumental Variable Is Utilization of Sleeve Gastrectomy in the 2 Years Prior to Treatment

	**Instrumental Variables Analysis**		**Difference (95% CI)**
	**Sleeve**	**Bypass**	
Mortality	0.88 (0.76-0.98)	1.85 (1.52-2.18)	-0.97 (-1.36, -0.58)
Complications	11.19 (10.52-11.85)	14.67 (13.48-15.86)	-3.48 (-5.18, -1.78)
ED Utilization	48.42 (46.92-49.92)	53.53 (52.34-54.72)	-5.11 (-7.50, -2.71)
Hospitalization	22.97 (21.93-24.03)	27.08 (25.65-28.51)	-4.10 (-6.20, -2.01)
Reintervention	8.60 (7.95-9.24)	12.37 (11.40-13.35)	-3.78 (-5.24, -2.31)
Revision	0.45 (0.29-0.60)	0.49 (0.31-0.67)	-0.04 (-0.36, 0.27)

Supplemental Table 2 Legend: The instrumental variables analysis was a 2-stage residual inclusion estimation method wherein the first stage was a multivariable logistic regression model to estimate the likelihood of undergoing sleeve gastrectomy (covariates: sleeve gastrectomy rate in the 2 years prior to treatment (the instrumental variable), age, sex, race and ethnicity, comorbidities, and year of surgery) and the second stage was a multivariable logistic regression model to estimate the absolute risk difference for each outcome (covariates: treatment, age, sex, race and ethnicity, comorbidities, year of surgery, and residuals from the first-stage regression model).

© 2022 Howard R et al. *JAMA Network Open.*
